# The attitudes of psychiatric hospital staff toward hospitalization and treatment of patients with borderline personality disorder

**DOI:** 10.1186/s12888-014-0380-y

**Published:** 2015-01-22

**Authors:** Ehud Bodner, Sara Cohen-Fridel, Mordechai Mashiah, Michael Segal, Alexander Grinshpoon, Tzvi Fischel, Iulian Iancu

**Affiliations:** Interdisciplinary Department of Social Sciences, Bar Ilan University, Ramat Gan, Israel; School of Education, Bar Ilan University, Ramat Gan, Israel; Tel Aviv University, Abarbanel Mental Health Center, Bat Yam, Israel; Technion, Shaar Menashe Hospital, Shaar Menashe, Israel; Tel Aviv University, Geha Mental Health Center, Petah Tikva, Israel; Tel Aviv University, The Beer Yaakov Mental Health Center, Yavne, Israel; The Yavne Mental Health Clinic, Dekel 4 st., Yavne, 81540 Israel

**Keywords:** Attitudes, Borderline personality disorder, Hospitalization, Mental health, Staff, Suicide

## Abstract

**Background:**

Negative attitudes towards patients with borderline personality disorder (BPD) may affect their treatment. We aimed to identify attitudes toward patients with BPD.

**Methods:**

Clinicians in four psychiatric hospitals in Israel (*n =* 710; psychiatrists, psychologists, social workers and nurses) were approached and completed questionnaires on attitudes toward these patients.

**Results:**

Nurses and psychiatrists reported encountering a higher number of patients with BPD during the last month, and exhibited more negative attitudes and less empathy toward these patients than the other two professions. The whole sample evaluated the decision to hospitalize such a patient as less justified than the decision to hospitalize a patient with Major Depressive Disorder. Negative attitudes were positively correlated with caring for greater numbers of patients with BPD in the past month and in the past 12 months. Nurses expressed the highest interest in studying short-term methods for treating patients with BPD and a lower percentage of psychiatrists expressed an interest in improving their professional skills in treating these patients.

**Conclusions:**

The findings show that nurses and psychiatrists differ from the other professions in their experience and attitudes toward patients with BPD. We conclude that nurses and psychiatrists may be the target of future studies on their attitudes toward provocative behavioral patterns (e.g., suicide attempts) characterizing these patients. We also recommend implementing workshops for improving staff attitudes toward patients with BPD.

## Background

Patients with Borderline Personality Disorder (BPD) are a challenge to the mental health system [1-3]. They engage negatively with the staff and have a bad reputation, create unconsciously hostility and violence, hurt themselves, threaten to suicide, antagonize the staff, drop out of treatment and even sue their therapists [4-6]. They are perceived as difficult, annoying, manipulative, and as “bad” and not “ill” [7,8]. Conceivably, the attitudes of mental health staff towards hospitalization and treatment of BPD patients may affect the way they deal with them and may escalate a vicious cycle of miscommunication and cause a revolving door of hospitalizations [9,10] and high dropout rates [11]. Our objective was to perform a comparative mapping of the attitudes of mental health teams toward BPD, between four professions (psychiatrists, psychologists, social workers and nurses), in four large psychiatric centers.

Numerous studies examined attitudes of mental health professionals toward BPD [5,12-20]. In a study with 516 Australian psychiatric staff members (i.e., nurses, psychiatrists, psychologists, social workers and occupational therapists), most respondents indicated difficulties posed by these patients (80%) and considered the interaction with these patients as more difficult than communicating with other groups of patients (84%) [21]. A study in Ireland with psychiatric nurses revealed similar findings [19]. Again, 80% viewed patients with BPD as more difficult to treat, while 81% believed that the care they receive is inadequate. Another study of attitudes of psychiatric nurses found more negativity and less sympathy and optimism toward patients with BPD, than toward schizophrenia or depression [20]. Nurses also perceived BPD patients as more dangerous and were more socially rejecting toward them. In a large survey with 706 American mental health clinicians (including psychiatrists, psychiatry residents, social workers, nurses, and psychologists), nearly half reported they preferred to avoid these patients [4]. The clinician's profession was significantly related to attitude, with nurses showing the lowest self-ratings on overall caring attitudes and social workers having the highest. Social workers and psychiatrists had the highest ratings on treatment optimism. Social workers and psychologists were most optimistic about psychotherapy effectiveness, while psychiatrists were most optimistic about medication effectiveness. Nurses had the lowest ratings on empathy toward patients with BPD and treatment optimism. Interestingly, in spite of the difficulty with these patients, caring attitudes, empathy, and treatment optimism were higher among staff that had cared for a greater number of patients with BPD in the past year [4].

A thorough psychometric enquiry into the counter-transference processes toward patients suffering from personality disorders (including BPD) was conducted by Betan et al. [22]. The researchers assessed clinicians’ cognitive, affective, and behavioral responses in interacting with these patients. For this purpose, they delivered a battery of instruments to a random sample of 181 psychiatrists and clinical psychologists in North America and aimed to develop a psychometric tool measuring counter-transference reactions toward patients with personality disorders. They found eight clinically and conceptually coherent counter-transference reactions (factors) that were independent of clinicians’ theoretical orientation and were associated in predictable ways with personality disorders. In regard to BPD, clinicians reported either over-involvement or flee (i.e., agreeing with items describing strong negative feelings, such as dread, repulsion). The authors concluded that counter-transference reactions are systematically related to patients’ personality pathology regardless of the clinicians' therapeutic approach. This emphasizes the strong impact that patients with severe personality disorders (including BPD) have on their clinicians [22].

In Israel, BPD patients are not recognized as suffering from mental illness, and due to the problematic nature of this disorder the hospitals' management does not see them as mandatory candidates for hospitalization, especially not lengthy ones [5]. They are usually admitted due to an acute crisis and after quick stabilization they are discharged to further community follow-up [Hirschman, personal communication]. The admission is usually to a closed ward and is voluntary, as in Israel compulsory hospitalizations are reserved only for psychotic patients who are also suicidal or aggressive. Furthermore, with the approaching 2015 Israeli Mental Health Reform [23], the Sick Funds that will become the service providers for ambulatory patients might not take full economic responsibility for these patients, especially as concerns their hospitalization (paid until now by the Ministry of Health). This may amplify the negative attitudes of mental health teams towards admission and treatment of these patients. Therefore, we thought that it would be important to evaluate staff attitudes toward patients with BPD in Israel.

In a previous study [5], we created two psychometric inventories for the measurement of cognitive and affective attitudes toward BPD patients. We also compared attitudes of different mental health professions toward patients with BPD. We showed that in comparison with psychiatrists and nurses, psychologists had less antagonistic judgments toward BPD and perceived them as less manipulative and rather eligible for hospitalization. Since we did not find a similar report in the literature, we interpreted this finding as deriving from the psychologists' attempt to accept, empathize and understand patients as opposed to the authoritarian and limit-setting styles of the other professions [24]. In line with the literature [25], all groups of practitioners perceived the tendency for suicide among these patients seriously and considered them as high-risk. However, our main finding, which is widely supported by the literature, was that nurses, being the professionals mostly exposed to continuous interactions with these patients, expressed less empathy, in comparison with psychiatrists and psychologists. This is in accordance with a recent review of studies on nurses' attitudes toward patients with BPD [3] that reported that nurses perceive patients with BPD as dangerous, powerful, unrelenting, and more difficult to take care of. These authors concluded that consequently, nurses respond to such patients with anger, social distance, less empathy, and more negative emotions and attitudes. Nevertheless, it is unclear whether nurses as a profession have worse attitudes towards BPD or whether the negative attitudes arise for example from more time spent with these patients on the ward or from the higher number of BPD patients treated by them.

Most studies conducted till now did not use valid and reliable measurement tools for the measurement of attitudes, did not use very large samples and did not sample many professions. We aimed to improve our original sample and to inspect if the nurses' tendency to express more negative attitudes toward BPD will also be evident in a new and much larger sample. In addition, we added a fourth group of clinicians (i.e. social workers) and used more sophisticated measurement tools of attitudes towards BPD.

In line with the reviewed literature we formulated three hypotheses:

1. Nurses would report more negative attitudes as compared with clinicians from the other professions (even after controlling for different employment and task variables).
2. All professions will report more negative attitudes and negative traits toward BPD than towards a patient with major depressive disorder (MDD) or generalized anxiety disorder (GAD).
3. Only nurses would attribute more negative traits to patients with BPD than to patients with MDD or GAD (interaction of profession × diagnosis).

## Methods

### Participants

The study was conducted on a proportional volunteers sample of mental health clinicians (n = 710) from four professions (psychiatry, psychology, social work, nursing) in four psychiatric hospitals. As more than half of the psychiatric beds are located at the center of Israel [26], we concentrated on this part of the country, by using a sampling method similar to the one used by Shapira and colleagues [27]. As in our previous study [5], participants were included in the study if they fulfilled the following criteria: (1) agreed to sign written informed consent to participate in the study after the procedure had been fully explained; (2) were older than 25 years; (3) had a minimal level of personal and professional experience (>1 year); (4) were certified psychiatrists, social workers, psychologists or nurses. Table 1 presents the distribution of background variables by profession.

**Table 1:** **Distribution of background variables by profession**

	**Profession**	**Psychiatry (n = 167)**	**Psychology (n = 162)**	**Social work (n = 100)**	**Nursing (n = 262)**	**Total (n = 691)**	**Statistical tests**
**Gender** ^**a**^							
	Female	76 (45.5%)	125 (77.2%)	84 (84.0%)	155 (59.6%)	440 (63.9%)	χ^2^ (3) = 56.39***
	Male	91 (54.5%)	37 (22.8%)	16 (16.0%)	105 (40.4%)	249 (36.1%)	
**Age** ^**b**^							
	*M*	46.78	40.79	43.49	47.41	44.62	*F*(3,650) =17.18***
	*SD*	9.90	10.19	9.67	9.34	9.78	
**Country of birth** ^**c,f**^							
	Israel	75 (48.1%)	128 (84.8%)	83 (85.6%)	117 (48.3%)	403 (62.4%)	χ^2^ (3) = 88.38***
	Abroad	81 (51.9%)	23 (15.2%)	14 (14.4%)	125 (51.7%)	243 (37.6%)	
**Family status** ^**d,f**^							
	In couple	138 (83.1%)	128 (81.0%)	79 (79.8%)	107 (82.5%)	452 (82.6%)	χ^2^ (3) = .60, *ns*
	Not in couple	28 (16.9%)	30 (19.0%)	20 (20.2%)	44 (17.5%)	122 (17.4%)	
**Seniority (years)** ^**e**^							
	*M*	16.45	11.05	14.66	21.15	15.83	*F*(3,665) =34.59***
	*SD*	11.28	9.46	9.94	9.39	10.02	
**Ward Type** ^**f**^							
	Closed	78 (49.1%)	42 (26.1%)	19 (19.2%)	189 (76.2%)	328 (49.2%)	χ^2^ (3) = 142.47***
	Not Closed	81 (50.9%)	119 (73.9%)	80 (80.8%)	59 (23.8%)	339 (50.8%)	

*Note*. ^**a**^Nineteen did not report their gender; ^**b**^Fifty-five did not report their age; ^**c**^Sixty did not report their country of birth; ^**d**^Thirty-five did not report their family status; ^**e**^Thirty-eight did not report their professional seniority; ^**f**^Country of birth, family status, and ward type were grouped into two categories. ***p < .001.

### Instruments

Participants completed two questionnaires measuring cognitive and emotional attitudes toward patients with BPD, and a questionnaire measuring attitudes either toward patients with BPD, patients with MDD or patients with GAD, using a short narrative. The cognitive attitudes inventory included 23 items from the original questionnaire [5], rated on a 5-point likert scare, ranging from 1 (strongly disagree) to 5 (strongly agree). A confirmatory factor analysis with varimax rotation on 21 items (two items were excluded because they could not discriminate between the factors and had low loadings) reconstructed three factors, similar to the original ones [5], that explained 39.54% of the total variance: (1) perception of suicidal tendencies (19.60% of variance, Cronbach's α = .75, e.g., "Death by suicide in BPD patients is very rare"); (2) need for hospitalization (12.28% of variance, Cronbach's α = .72, e.g., "a legitimate reason for hospitalizing a BPD patient is rehabilitation"); (3) antagonism (7.66% of variance, Cronbach's α = .63, e.g., "when a BPD patient demonstrates fears, auditory hallucinations, and suicidal threats, the secondary gain should be diagnosed and treated accordingly"). Three scores based on the means of responses of each factor were computed for each participant. Higher scores reflected a perception that the patient was more problematic.

The emotional attitudes inventory was similar to the original questionnaire [5] and included 20 items, rated on a 5-point level of agreement likert scare, ranging from 1 (strongly disagree) to 5 (strongly agree). Two items were excluded because they could not discriminate between factors and had low loadings. A confirmatory factor analysis with varimax rotation on 18 items reconstructed three factors, similar to the original ones, that explained 48.54% of the total variance: (1) negative affect (28.70% of the total variance, Cronbach's α = .86, e.g., "I feel angry when a BPD patient threatens to commit suicide"); (2) difficulty to treat the patients (12.54% of the variance, Cronbach's α = .67, e.g., "It is easier for me to treat schizophrenic patients than BPD patients"); (3) lack of empathy (three reversed items, 7.29% of the total variance, Cronbach's α = .64, e.g., "I feel empathy towards BPD patients"). Three scores based on the means of responses of each factor were computed for each participant, with higher scores reflecting negative emotional attitudes.

The third attitudes questionnaire was an implicit attitudes assessment and included a vignette describing a suicide attempt made by a woman in her middle twenties, a relatively long time of hospitalization (three months), a relapse of suicidal ideation after discharge and a recurrent arrival of the patient to the emergency room. This clinical pattern is in line with the pattern of recurrent suicidal ideations and suicidal behaviors in BPD [25], but may also be part of MDD or anxiety disorder [28,29]. The exposure of clinicians to clinical descriptions of patients followed by a request to evaluate the patients had already been used as a way to comparatively measure clinicians' attitudes toward BPD [18]. However, unlike the abovementioned study, in the current study, while all the participants were exposed to the same narrative, about a third (n = 244) provided their answers in response to a vignette with a given diagnosis of BPD, about a third (n = 214) in response to a vignette with a diagnosis of MDD, and about a third (n = 209) in response to a vignette with a diagnosis of GAD. Since participants didn't know that their evaluations of patients with BPD are being compared with evaluations of patients with other diagnoses, we regard this as an implicit measure.

After reading the vignette, participants were asked to rate their evaluations on a 7-point differential semantic continuum, ranging from one pole (scored 1) to the other (scored 7), and to report if the decision to hospitalize the patient was justified or unjustified, correct or wrong, reasonable or unreasonable, professional or not professional, and effective or ineffective. Internal reliability for this five-item measure was high (Cronbach's α = .89). Hence, the mean of these five items was computed for each participant. The higher the score, the more negative was the attitude toward the decision to hospitalize. Participants were also asked to evaluate if a three months hospitalization is a reasonable or an unreasonable decision. The higher the score, the more negative was the attitude toward the length of hospitalization. In addition, we measured the participants' evaluations of the quality of treatment the patient received, using the same items and the same differential semantic scale. Internal reliability was high (Cronbach's α = .86). The mean of these five items was computed for each participant. The higher the score, the more negative was the attitude toward the quality of treatment the patient received. Moreover, participants were asked to evaluate 13 traits of the patient (e.g., cooperative-uncooperative, adaptive-not-adaptive, wise-stupid, selfish-unselfish, manipulative-non-manipulative, pleasant-unpleasant, bad-good, reasonable-unreasonable) based on the vignette, on a similar 7-point differential semantic continuum. The scales and some of the traits were based on previous studies that measured acceptability of suicide [30]. Scores were computed for each trait separately and the higher the score, the more negative was the attitude (three items were reversed). Finally, clinicians provided details about their personal background (e.g., gender, age, seniority), and about their experience with BPD patients (e.g., years of experience with these patients, familiarity with therapies for BPD, and interest in studying therapies for BPD).

### Procedure

The study was approved by the institutions' review boards (Beer Yaakov, Abarbanel, Geha and Shaar Menashe Hospitals). Research assistants approached all the professionals meeting the abovementioned criteria, while serving a shift on the ward. Using the study informed consent document, the research assistant explained the purpose of the study and gave the questionnaires to the subjects. The participants filled out the questionnaires either in the presence of the research assistant, or later on. Completed questionnaires were then placed together with other completed questionnaires in order to ensure anonymity. Return rate of questionnaires was much higher than that reported in other attitudes studies of psychiatric personnel [31-33] and ranged between 40.91% in one of the hospitals to 70.5% in another. The relative representation of participants from each profession was also high, ranging between 49.05% (nursing) to 75.6% (social work). Data were collected between July 2012 and September 2013.

### Statistical analysis

Statistical analysis was performed with SPSS version 20.0. Multivariate Analyses of Covariance (MANCOVAs) were conducted in order to compare the four groups of clinicians on each of the three factors of cognitive attitudes and emotional attitudes. Background variables that significantly differed between the four groups (i.e., gender, seniority and ward type, see Table 1) were controlled (except for age, which highly correlated with seniority, r = .83, p <. 001, and is less relevant than seniority for clinicians' attitudes toward the patients). MANOVAs were also conducted in order to compare the attitudes of participants on several dependent variables (i.e., the correctness of the decision to hospitalize, the appropriateness of the length of hospitalization, the quality of treatment, and the 13 traits) between professions and toward patients with BPD/ MDD/ GAD. Discriminant analysis, which is a multivariate technique that identifies the combination or combinations of variables that best separate groups [34] was also conducted. The purpose of this analysis was to provide an integrative view of the unique and combined effects of the cognitive and emotional attitudes toward BPD in differentiating between the four professional groups and between the three diagnoses. Moreover, the association between number of patients with BPD treated in the last month and last 12 months with the negativity of attitudes questionnaires' scores was also calculated. Additional analyses examined the comparative distribution of skills in treating BPD patients, and of training needs reported by the four professions. Differences were also evaluated using Univariate analyses of variance and Chi-square test with continuity correction. Clinicians rated their level of interest in learning short and long-term methods for treating patients with BPD, on a scale from 1 (low interest) to 5 (high interest). They were required to report if they had an interest (yes/no) in improving their skills.

## Results

**Figure 1:**
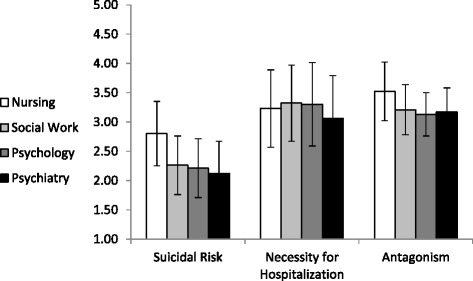
**Means and standard deviations of cognitive attitudes toward patients with BPD by profession.**

In line with the first hypothesis, nurses reported more negative cognitive attitudes toward patients with BPD than the other three professions. Figure 1 presents means and standard deviations for the three cognitive attitude factors, namely suicidal risk, necessity for hospitalization and antagonism.

As can be seen from Figure 1, nurses (according to Scheffe post-hoc tests) provided higher evaluations of the suicidal risk (M = 2.80, SD = 0.55 vs. social workers, M = 2.26, SD = 0.50; psychologists, M = 2.21, SD = 0.50, and psychiatrists, M = 2.12, SD = 0.55) and more antagonistic attitudes toward patients with BPD, ps < .001, (M = 3.52, SD = 0.50 vs. social workers, M = 3.21, SD = 0.43; psychologists, M = 3.13, SD = 0.37, and psychiatrists, M = 3.17, SD = 0.41). Moreover, nurses (M = 3.23, SD = 0.66) and psychiatrists in particular, (M = 3.06, SD = 0.73) provided lower scores regarding the necessity for hospitalization, as compared with psychologists (M = 3.30, SD = 0.71) and social workers (M = 3.32, SD = 0.65), ps < .05. In addition, as shown in Figure 1, the general evaluation of suicidal risk regarding patients with BPD is low (means are located at the lower part of the scale), and the negative evaluations provided in regard to necessity for hospitalization and antagonism are relatively high (means in upper part of the scale).

The means and standard deviations presented in Figure 1 were computed after main effects were found for profession regarding the three cognitive attitude factors: suicide risk, F(3,674) = 69.64, p < .001, η^2^ = .24; necessity for hospitalization, F(3,674) =4.29, p < .01, η^2^ = .02; and antagonism, F(3,674) = 34.59, p < .001, η^2^ = .14. The covariates (i.e., gender, seniority and ward type) were not significant (p > .05) and had no effect on the three cognitive attitude factors. Moreover, findings were similar when the Multivariate analyses of variance were reconstructed without the covariates. Furthermore, the differences in cognitive attitudes between the professions remained significant, even after controlling the level of specific education about BPD patients, the number of BPD patients treated last month, the number of BPD patients treated last year, the reported level of knowledge about Dialectical behavior therapy (DBT), and the reported level of practice with BPD patients, by entering these variables as covariates into the analysis (in addition to gender, seniority and ward type).

**Figure 2:**
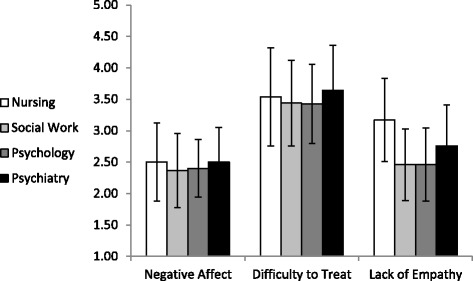
**Means and standard deviations of emotional attitudes toward patients with BPD by profession.**

In line with the first hypothesis, nurses also reported more negative emotional attitudes toward patients with BPD, than the other three professions. Figure 2 presents means and standard deviations for the three emotional attitude factors, namely: difficulty to treat, negative affect and lack of empathy.

As can be seen from Figure 2, nurses (according to Scheffe post-hoc tests) reported more lack of empathy toward patients with BPD, as compared with psychologists (M = 3.17, SD = 0.66 vs. M = 2.46, SD = 0.58) and social workers (M = 2.46, SD = 0.57), p < .001, but not in comparison with psychiatrists (M = 2.76, SD = 0.65), who also reported more lack of empathy than psychologists (p < .001) and social workers (p < .01). In addition, as shown in Figure 2, the general evaluations of negative affect and lack of empathy regarding patients with BPD are less prominent (means in lower part of the scale, except for lack of empathy reported by the nurses).

The means and standard deviations presented in Figure 2 were computed after main effects were found for profession in respect to two out of three emotional attitude factors, i.e., difficulty to treat, F(3,662) = 2.71, p < .05, η^2^ = .01, and lack of empathy, F(3,662) = 54.04, p < .001, η^2^ = .20. There were no significant differences in regard to negative affect, F(3,662) =1.96, p = .12, η^2^ = .01. The covariates (i.e., gender, seniority and ward type) were not significant (p > .05) and had no effect on the three emotional attitude factors. Moreover, findings were similar when the Multivariate analyses of variance were reconstructed without the covariates. Furthermore, the differences in emotional attitudes between the professions remained significant, even after controlling the level of specific education about BPD patients, the number BPD patients treated last month, the number of BPD patients treated last year, the reported level of knowledge about DBT, and the reported level of practice with BPD patients, by entering these variables as covariates to the analysis (in addition the gender, seniority and department type). In general, nurses exhibited more negative attitudes toward patients with BPD, and thus the first hypothesis was confirmed.

Discriminant analysis included variables (cognitive and emotional attitudes toward BPD patients with covariates) and these were entered in simultaneous fashion. Focusing on attitudes measures that discriminated between the four professional groups, the discriminant function reflected mainly these attitudes. Positive and negative standardized canonical discriminant function coefficients reflect more and less negative attitudes, respectively. Table 2 presents the results of the discriminant analysis, examining overall attitudes toward BPD patients by profession.

**Table 2:** **Discriminant analysis between the four professional groups**

**Functions at group centroids**		
	**Function 1**	**Function 2**
Psychiatry	1.177	.150
Psychology	-.830	.428
Social work	−1.004	.272
Nursing	-.287	-.750
**Standardized canonical discriminant function coefficients**		
	**Function 1**	**Function 2**
Negative affect	-.096	-.209
Difficulty to treat	.109	-.290
Empathy	**-.464**	.148
Suicidal risk	**.316**	**.533**
Necessity for hospitalization	.000	**.311**
Antagonism	**.355**	.209

*Note. N* = 628 after a listwise deletion of cases with missing data. Entries present results of a simultaneous solution. Coefficients of 0.30 or above appear in boldface type.

The analysis yielded three functions, but only two were significant. As can be seen from Table 2, the profession of nursing received the highest score (1.18) on that function and the professions of psychology and social work received the lowest scores on the first function (−1.00, and – 0.83, respectively). Specifically, the difference between nurses and psychologists (and social workers) on the first function associated nurses with more difficulty to treat, less empathy, higher evaluation of suicidal risk, and with more antagonism. In regard to the second function, the profession of social work received the highest score (0.43), and the profession of psychiatry received the lowest score (−0.75). Specifically, the difference between social workers and psychiatrists on the second function associated social workers with less negative affect, with less experienced emotional treatment difficulties and with more empathy, but also with a higher evaluation of suicidal risk, with higher evaluations regarding the necessity for hospitalization and with more antagonism.

In line with hypothesis 2 that participants would report more negative attitudes toward patients with BPD than toward patients with MDD or GAD, a main effect of diagnosis was found in regard to justification of the decision to hospitalize a patient with BPD, F(3,648) = 6.06, p < .01, η^2^ = .02, the length of three months hospitalization, F(3,648) = 6.17, p < .01, η^2^ = .02, and the quality of treatment in hospital, F(3,648) = 3.38, p < .01, η^2^ = .01. The hospitalization of a patient with BPD was perceived as less justified than that of a MDD patient (M = 5.07, SD = 1.50 vs. M = 5.55, SD = 1.34, respectively, p < .05), but did not significantly differ from a GAD patient (M = 4.97, SD = 1.48, respectively, p > .05). In contradistinction to our second hypothesis, Scheffe post-hoc tests revealed that it was the patient with GAD, whose 3-month length of hospitalization was perceived as less justified than of a patient with MDD (M =3.59, SD = 1.66 vs. M =4.07, SD = 1.5, p <. 05), and not a patient with BPD (M = 3.76, SD = 1.76). Scheffe post-hoc tests did not reveal significant differences in the estimation of quality of treatment in hospital between diagnoses.

Discriminant analysis included three measures (justification of the decision to hospitalize, evaluation of treatment, and negativity of traits) which were entered in simultaneous fashion. Focusing on these measures that discriminated between the three diagnoses, the discriminant analysis yielded two functions, but only one function was significant (*p* < .01). Regarding functions at group centroids, the vignette which described a BPD patient received the lowest score on that function (−0.14) and the vignette which described a MDD patient received the highest score (0.23). Specifically, the difference between the two vignettes positively associated BPD patients with an unjustified decision to hospitalize the patient, with more negative evaluation of the treatment, and with more negative traits (the standardized canonical discriminant function coefficients were respectively 0.81, 0.25 and 0.29).

Another main effect was found for profession in regard to the justification of the decision to hospitalize the patient, F(3,648) = 6.30, p < .001, η^2^ = .03. Nurses rated the decision to hospitalize the patients as more justified (M = 5.55, SD = 1.50), in comparison to social workers (M = 5.10, SD = 1.47), psychologists (M = 5.02, SD = 1.23), and psychiatrists (M = 5.07, SD = 1.50), but not in respect to the 3-month length of hospitalization, or the quality of treatment (p > .05). Moreover, an interaction of diagnosis × profession was found in regard to length of hospitalization, F(6,648) = 6.79, p <. 05, η^2^ = .02, but post-hoc analyses revealed it was only in regard to GAD. Finally, a main effect was found regarding four of the traits (we used Bonferroni correction due to numerous comparisons), which supported the second hypothesis that patients with BPD are perceived more negatively than patients with other diagnoses (according to scheffe post-hoc tests to MDD, but not GAD). Relevant findings are presented in Table 3.

**Table 3:** **Means, standard deviations and statistical comparisons of traits attributed to the three diagnoses**

**Trait**	**BPD**	**MDD**	**GAD**	**Statistical tests**
Selfish	4.19 (1.06)	3.94 (1.29)	4.31 (1.25)	F(2,531) =4.67**, η^2^ = .02
Manipulative	4.36 (1.20)	4.14 (1.35)	4.55 (1.42)	F(2,531) =4.40*, η^2^ = .02
Dramatic	4.79 (1.31)	4.52 (1.29)	4.93 (1.29)	F(2,531) =4.69**, η^2^ = .02
Passive	3.66 (1.41)	3.87 (1.31)	3.54 (1.20)	F(2,531) =3.13*, η^2^ = .01

*Note*. BPD=Borderline personality disorder; MDD=Major depressive disorder; GAD=Generalized anxiety disorder; Standard deviations are presented in parentheses; *p < .05, **p < .01.

As seen in Table 3, higher means of negative traits were reported for patients with BPD than for patients with MDD. To sum up, regarding the second hypothesis we found that the decision to hospitalize a patient with BPD was perceived more negatively than the decision to hospitalize a patient with MDD and the attribution of negative traits to patients with BPD was stronger than the attribution of negative traits to patients with MDD (but not than the decision to hospitalize a patient with GAD or the traits attributed to this patient). Therefore, the second hypothesis was partially supported.

In contradistinction to the third hypothesis that nurses only would attribute more negative traits to patients with BPD than to patients with MDD or GAD, no significant interactions of profession × diagnosis were found in regard to the negative traits' evaluation. Nurses did not have especially negative traits' evaluation toward patients with BPD, as compared with patients with MDD or GAD. Therefore, no discriminant analysis was conducted.

### Supplementary analyses

Supplementary analyses include findings regarding the professional characteristics of the studied professions relevant to their attitudes toward BPD patients. Table 4 presents these characteristics.

**Table 4:** **Means, standard deviations, frequencies and percent, and statistical comparisons of supplementary analyses**

**Profession**	**Nurs.**	**SA**	**Psyh.**	**Psych.**	**Statistical tests**
BPD last month	3.67 (3.94)	1.84 (2.68)	1.81 (2.09)	2.95 (4.21)	*F*(3,372) =10.18***, η^2^ = .08
BPD last 12 months	11.30 (17.56)	3.02 (3.48)	3.44 (4.14)	17.44 (35.95)	*F*(3,372) =10.33***, η^2^ = .08
Knowledge in DBT	2.01 (1.07)	2.13 (1.22)	2.07 (1.06)	2.43 (1.09)	F(3,635) =5.02**, η^2^ = .02
Interest in short-term	4.05 (0.90)	3.99 (1.07)	3.94 (0.97)	3.74 (1.12)	F(3,652) =3.36*, η^2^ = .02
Interest in long-term	3.85 (1.04)	4.08 (1.01)	4.15 (0.79)	3.61 (1.12)	*F*(3,652) =8.83**, η^2^ = .04
Interest in therapy	234 (93.2%)	95 (96.0%)	150 (94.9%)	145 (87.9%)	χ2 (3) = 12.58**
Interest in diagnostic	226 (89.7%)	89 (89.9%)	133 (84.7%)	118 (71.5%)	χ2 (3) = 8.63*

*Note*. Nursing=Nurs. Social work**=**SA**;** Psychology**=**Psyh; Psychiatry=Psych. The first two rows are numbers of BPD patients treated last month or last 12 months; The next five rows are reports on knowledge in DBT, interest in learning short-term therapy and long-term methods for patients with BPD, interest in improving therapeutic and diagnostic skills; Standard deviations are presented in parentheses/percent; DBT = Dialectic Behavior Therapy; *p < .05, **p<. 01, ***p < .001.

As shown in Table 4, the mean number of patients with BPD treated by nurses during the last month was higher than the mean number of the other three professions, and Scheffe post-hoc tests revealed that nurses (but also psychiatrists) significantly encountered with the highest number of patients with BPD (p < .01). Scheffe post-hoc tests also revealed that psychiatrists reported a higher level of knowledge in DBT [35], than nurses and psychologists (p < .05). Nurses reported a higher level of interest than psychiatrists in studying short term psychotherapy for BPD (p < .05), and psychologists reported a higher level of interest than nurses in studying long term psychotherapy for BPD (p < .01). Although the percent of participants that expressed interest in improving their therapeutic and diagnostic skills was very high, in comparison to the other professions, psychiatrists significantly expressed less interest in improving their psychotherapeutic and diagnostic skills with these patients.

Finally, we computed correlations between some variables presented in Table 4 and the attitudes of the staff toward these patients. A positive correlation was found between the number of patients with BPD treated during the last month and negative affect toward these patients (r = .13, p < .01) and also between the number of patients treated during the last 12-month and negative affect (r = .13, p < .01). The higher negative attitudes, the greater the number of patients with BPD that were treated. However, when dividing the data according to profession, the correlation strengthened among nurses for both the number of patients with BPD treated during the last month (r = .26, p = .001), and for the number of patients treated during the last 12-month (r = .30, p < .001) and became negative for the other three professions: social workers (r = −.25, p < .05) for the number of patients with BPD treated during the last month, psychologists: (r = −.22, p = .01) for the number of patients with BPD treated during the last month, and for number of patients treated during the last 12-month : (r = −.20, p < .05), and psychiatrists (r = −.27, p < .01) for the number of patients treated during the last 12-month.

## Discussion

Our main findings include the following: 1) nurses exhibited more negative cognitive attitudes toward patients with BPD and less empathy than psychologists and social workers, but not in comparison with psychiatrists. 2) the inadequacy of the decision to hospitalize a patient and the attribution of negative traits were more prominent for a patient with BPD than for a patient with MDD. 3) nurses did not attribute more negative traits to patients with BPD than to patients with MDD or GAD, as opposed to the other three professions. 4) nurses and psychiatrists reported that they encountered a significantly higher number of patients with BPD during the last month, nurses expressed the highest interest in studying short term methods for treating patients with BPD, and a lower percentage of psychiatrists expressed an interest in improving their therapeutic and diagnostic skills for BPD.

We used valid questionnaires and a large sample of clinicians from four professions in the field of mental health. We found significant differences on the attitudes towards BPD between the four professions. Nurses reported more negative attitudes (more feared suicidal risk of patients with BPD, more antagonism and less empathy towards patients with BPD), but not more negative perception of traits of BPD patients on the implicit attitudes inventory. Nurses indeed had been found to regard BPD patients as being more in control of negative behavior than patients with schizophrenia or depression [18] and attributions of control were inversely related to staff sympathy. Indeed, in the large study by Black et al. [4], psychiatric nurses had the lowest ratings on overall caring attitudes toward patients with BPD and also had the lowest ratings on empathy. However, we agree with the stipulation by Sansone and Sansone [3] that the attitudes nurses present simply reflect a very human reaction to the complex and pathological behaviors of these patients.

Although we reconfirmed the higher negative attitudes among nurses which were found in the abovementioned studies, as well as by our research group [5], we also found that as compared to psychologists and social workers, the psychiatrists also held negative attitudes, and especially did not as much support the admission of these patients. Nurses and psychiatrists encounter more BPD patients and this explains their higher negative attitudes (compared to psychologists and social workers).The psychiatrists are in charge of admissions and discharges, and this responsibility is difficult to implement with BPD patients who tend to present with exacerbations during the discharge process [36]. The nurses, the boundary-keepers on the ward, interact intensively with these patients on an hourly basis. This intense interaction may result in less empathy and more antagonistic attitudes. Interestingly, while in our previous study, nurses reported significantly less empathy and a similar level of antagonism toward BPD patients [5], in the present study the difference between nurses and psychiatrists in the level of lack of empathy did not reach significance, and nurses exhibited a higher level of antagonism toward BPD patients (the differences between these two professions and psychologists were reconstructed). Since the current study is based on a much larger and representative sample (710 vs. 57 clinicians), we believe that the current findings are more reliable. Unlike nurses, psychiatrists are not required to be in a continuous and direct contact with these patients. Moreover, psychiatrists receive more medical education and have more clinical experience, as compared with nurses. Therefore, psychiatrists can cognitively balance their attitudes toward BPD patients and demonstrate less antagonistic attitudes. However, they may still find it difficult to regulate their emotions toward these patients whom they have to hospitalize and who suffer from a personality disorder, and not from a clear-cut psychiatric disorder. Therefore, similar to nurses, they report lack of empathy toward these patients. Interestingly, despite the report by nurses of less empathy and more antagonism, the nurses did not perceive these patients as "bad" in comparison with the other professions or in comparison to other diagnoses. Nurses understand the challenges posed by patients with BPD and also understand that they are less trained to tackle these challenges. Indeed, the nurses expressed interest in learning short-term interventions. One can understand this need because their training is "medical" and is not affected by ideas of long psychological processes (a prevalent issue in psychology and social work). Moreover, while their training in personality disorders is the least profound as compared to the other professions, they have to manage larger numbers of patients with BPD and their contact with them is more intensive. It is reasonable that as compared to the other professions, the suffering of these patients is seen differently by psychiatrists as it is not based on a disease model. Also, as physicians, psychiatrists have the highest responsibility for the safety of their patients, and are more exposed to lawsuits than the other professions [37,38]. Hence, they may become more defensive and thus report more distance and less empathy towards these patients. As these patients do not fit the medical model, psychiatrists express less interest than the other professions in improving both their psychotherapeutic and diagnostic skills with BPD. Another explanation is that they are indeed better trained in these topics.

Why do nurses have higher negative attitudes towards BPD patients? Is this a result of their profession, or because they spend more time with them? Is it because they know less about the personality psychopathology and therefore are having a harder time mentalizing these patients? In our analyses, the differences between the professions remained significant, even after controlling for such alternative explanations, and this may imply that their attitudes can be related merely to their profession. Future studies can examine more specifically what attributes of their profession are related to their attitudes.

In line with the findings of Black et al. [4] who showed that social workers had the highest overall caring attitudes and also the highest treatment optimism (with psychologists also regarding the effectiveness of psychotherapy), we found that social workers reported higher evaluations than nurses and psychiatrists regarding the necessity of hospitalization, and expressed the lowest level of lack of empathy (i.e. more empathy) toward these patients (similar to psychologists). However, we did not find any differences between social workers and psychologists. This gap might evolve from the different methodology used in our study as compared to the study by Black et al. [4]. Nevertheless, Black and colleagues [4] showed that social workers and psychologists were more likely to endorse the value of psychosocial treatments. It is possible that this derives from the basic training of both professions that emphasizes the principles of acceptance, empathy and understanding as opposed to the authoritarian and limit-setting styles of the other professions [24].

Another finding in our study requires attention. We found a positive correlation between the number of patients with BPD treated during the last month or during the last 12 months and negative affect toward BPD. This is in contradistinction with the findings of Black et al. [4] who found the opposite. Black et al. [4] indeed mentioned that they were unable to determine whether treating more patients with BPD positively affects one's attitudes or whether clinicians with positive attitudes are more likely to care for more patients by personal preference (the chicken or the egg causality dilemma). However, when dividing our data by professions, we found that only nurses demonstrated positive correlation between the numbers of BPD patients treated and negative attitudes toward these patients, whereas the other professions demonstrated a negative correlation between the two. In other words, while treating more patients with BPD was correlated with more negative attitudes toward these patients among nurses, it was correlated with less negative attitudes among the other professions. Therefore, it is possible that either because nurses are less able to choose to treat patients with BPD or because they have to encounter more BPD patients, the more patients with BPD they encounter the more negative they become. However, because the other three professions can either chose the number of patients they have to treat, or because they treat less patients, the more patients they treat, the less negative they become. Future studies can provide answers to these findings.

The issue whether these patients should be fully hospitalized has not been resolved in the literature. Many recommend refraining from hospitalizing these patients [39-41], especially when the aim is to prevent suicide. The treatment method that is more efficacious includes partial hospitalization and short residential stays with community treatment with DBT. In Israel there is no uniform policy regarding the admission to psychiatric wards and moreover, while DBT is a more and more accepted treatment method, most therapists are not well acquainted with this method. Thus, a residential system with unclear policy and a community setting with lack of expertise in DBT (and lack in general resources) create together a vacuum in this topic. In real life, these patients are hospitalized in Israel due to crisis situations and many of these situations include suicide tendencies.

Our study has several limitations, such as a possible report bias concealing negative attitudes and a participation bias holding that those who participated in our survey held different attitudes as compared to those who did not participate. However, since our participation rates were very high (40.71% to 70.5%), we consider the sample as representative and regard our findings as generalizable.

Because attitudes influence treatment outcome, it is crucial that clinicians learn to counter negative attitudes [4]. The first step in this attempt is to become aware of those emotions aroused by patients with BPD and also to achieve awareness of the distress caused by BPD and to the low self-esteem associated with BPD [42]. A study with 271 staff members [42] showed that a one-day workshop was efficient in the improvement of problem-solving and understanding of the emotional state of these patients. The workshop increased empathy towards these patients and reduced hostility on the part of the staff and also increased coping ability to work with them. We recommend that workshops with psychiatrists and nurses should be implemented in our hospitals, together with DBT training [6,35]. Training can be based on effective therapeutic methods for treating patients with BPD [43,44], such as Transference-focused therapy [45], Mentalization-based treatment [46], Schema therapy [47] and DBT [48]. The implementation of our recommendations might improve attitudes toward BPD and the treatment delivered.

## Conclusions

How can the abovementioned negative attitudes be changed? We previously showed that all practitioners supported the idea that the treatment of hospitalized BPD patients should combine emotional support, containment, psychotherapy and medications [5]. Thus, although many clinicians have negative opinions about BPD, they still acknowledge its complexity and realize the need for different disciplines to combine efforts in treating BPD. It is important to train all sectors in DBT, but we recommend to reduce the burden of treatment of these patients and to distribute it more equally between the four sectors (nowadays the burden is on nurses and psychiatrists).

## Abbreviations

BPD: Borderline personality disorder
MDD: Major depressive disorder
GAD: Generalized anxiety disorder
MANCOVA: Multivariate analyses of covariance
MANOVA: Multivariate analysis of variance
DBT: Dialectical behavior therapy
